# The Interplay of Oxidative Stress, Mitochondrial Dysfunction, and Neuroinflammation in Autism Spectrum Disorder: Behavioral Implications and Therapeutic Strategies

**DOI:** 10.3390/brainsci15080853

**Published:** 2025-08-11

**Authors:** Ansab Akhtar, SK Batin Rahaman

**Affiliations:** 1 LSU Health, Neuroscience Center of Excellence, School of Medicine, New Orleans, LA 70112, USA; 2 School of Pharmacy, Jamia Hamdard University, New Delhi 110062, India; neospectator@gmail.com

**Keywords:** autism spectrum disorder, oxidative stress, mitochondrial dysfunction, neuroinflammation, speech and language impairment

## Abstract

Autism spectrum disorder (ASD) deals with several symptoms, including language and speech impairment and developmental delays. The main brain regions affected could be the prefrontal cortex (PFC) or the temporal lobe. The detrimental features could include oxidative stress, mitochondrial dysfunction, and neuroinflammation. Most often, these phenomena are interrelated and can lead to one another, creating a vicious cycle. They also influence the regulation of certain genes involved in the pathogenesis of ASD or related behavior. In the brain regions prone to these detrimental features, a cascade of free radicals, inflammatory cytokines, and mitochondrial energy disruptions is initiated. These actions during the prenatal or developmental stage of the child potentially lead to ASD symptomatic features, such as social isolation, communication difficulty, speech and language impairment, cognitive dysfunction, and intellectual disability. The more recent theories, including genetics, epigenetics, and the gut–brain axis, have been demonstrated to play a greater role in ASD pathology, often being associated with the more common ones as mentioned above. We also introduced some of the neurological disorders possessing shared genetic and behavioral traits with ASD. Many genes playing a role in ASD-like features and their potential targeted drugs were explained briefly. However, there are limited therapeutic options, and molecular pathways related to this disorder are less explored. Currently, researchers and therapists are racing to uncover a concrete remedy. This review also provides a brief outline of potential antioxidant, mitochondrial, and anti-inflammatory therapies. We finally included some novel strategies to diagnose and manage autistic pathology and symptoms.

## 1. Introduction

Autism spectrum disorder (ASD) encompasses a range of medically diagnosed disorders that are said to result from impaired social interactions and communication, as well as the repetitive performance of certain behaviors and interests. Children with autism commonly experience multiple speech and language impairments, which are one of the greatest barriers faced, greatly affecting learning, sociability, and quality of life [1]. Many interacting pathways contribute to these communication deficits in ASD, such as genetic, environmental, and biological mechanisms [2]. Recently, it has been shown that oxidative stress is one of the most relevant biological mechanisms that may affect the pathophysiology of ASD or its subtypes [3,4,5]. The oxidative stress mechanism relates to an imbalance between reactive free radical molecules that can damage cells and the antioxidant systems of the organism. This mechanism can lead to oxidative injury in cells and tissues, such as neurons, affecting neurodevelopmental processes [6]. Children with ASD are found to have greater levels of oxidative stress markers, such as lipid and DNA damage biomarkers, when compared with neurotypical individuals. This signifies that these children are more prone to an enhanced state of neurodevelopmental complications in comparison to neurotypical children [7]. ASD-related speech and language areas for the typical speech, motor, and language centers, which are of great use for communication and sociability, involve many brain regions such as the right temporal, right parahippocampal gyrus, ventral striatum, limbic areas, putamen, and entorhinal cortex [8]. These regions, when exposed to oxidative stress, can affect the ASD brain regions. Oxidative stress has been an apparent issue in the pathogenesis of ASD, especially in compromised neuronal signaling, mitochondrial dysfunction, and neurogenesis. These neurobiological imbalances may directly interfere with key areas of the brain that are concerned with speech and language processing, such as the prefrontal cortex (PFC) and the temporal lobes. According to empirical data, the disruption of neurotransmitter homeostasis, synaptic plasticity, and maintenance of mitochondrial energy output, which are central to neurodevelopmental activities, including communication and language acquisition, are diminished by increased oxidative stress [3,9]. The usefulness of oxidative stress as a diagnosis and treatment target is also supported by the levels of related markers such as elevated levels of malondialdehyde (MDA) and 8-hydroxy-2′-deoxyguanosine (8-OHdG) and reduced glutathione (GSH) levels in patients with ASD [10,11]. Early studies of specific antioxidant treatment have shown beneficial effects on oxidative damage and cognitive and behavioral outcomes in ASD children, with only limited consistency in positive clinical effects, hence needing further thorough investigations. The significance of this discovery is the importance of further research into oxidative stress as not only a biochemical indicator, but rather, as a mechanism pathway that leads to language and communication deficits in autism.

**Figure 1:**
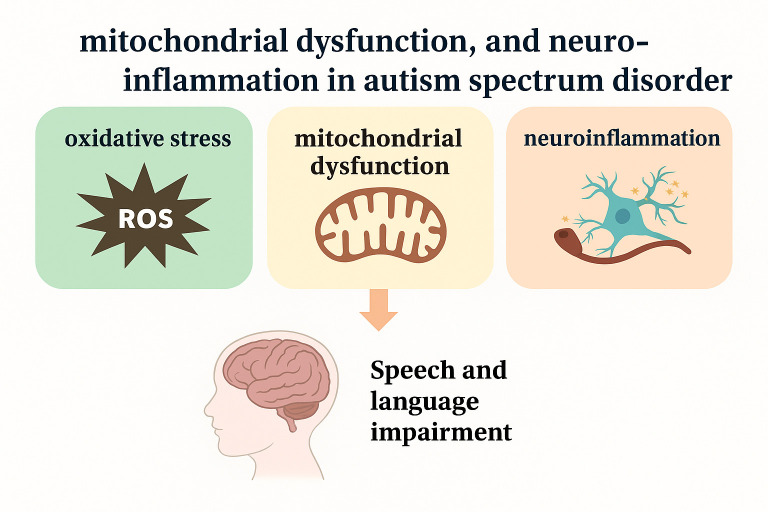
Reactive oxygen species (ROS), mitochondrial dysfunction, and neuroinflammation decrease the functionality of the corticolimbic pathways that play a vital role in communication and language comprehension.

Oxidative stress has been reported to generate abnormalities in neuronal signaling, mitochondria, and new cell generation in the brain [12], which are important elements for proper communication and language learning [13]. Analyzing oxidative stress as a reason for language and speech conditions in children with autism can help define biological markers for easy diagnosis of the condition and a way to help treat the condition. For example, using antioxidants would help alleviate the negative effects caused by oxidative stress in aiding healthy brain development. This review paper deals with the problems and importance of oxidative stress in children with autism, in particular, its influence on speech and language development. This work aims to sensitize parents, administrators, and other stakeholders about oxidative stress as an associated factor in speech and language problems in children with ASD and emphasizes that more research should be performed in this area. Furthermore, a highly related phenomenon, mitochondrial dysfunction, will also be elaborated in the context of ASD-related oxidative stress. Additionally, peripheral and central-generated inflammation can also be a contributing factor in ASD pathophysiology. Moreover, our review collectively presents the role of these detrimental features (oxidative stress, mitochondrial dysfunction, etc.) in connection with autism, where they are interconnected rather than having individual roles. Moreover, speech and language impairments are also one of the main focuses of this review (Figure 1). Speech impairment is one of the features in autistic children, other than intellectual and social distortions. Additionally, the phenomena of oxidative stress, mitochondrial dysfunction, and neuroinflammation are more notoriously associated with neurodegenerative diseases such as Alzheimer’s disease and Parkinson’s disease rather than with neurodevelopmental disorders such as autism, which has not been as widely researched as compared to the neurodegenerative diseases in the context of these features.

## 2. Oxidative Stress in Autism Spectrum Disorder

Numerous investigations that have been conducted in the last ten years across the globe have been performed to investigate oxidative stress and its correlation with the changes in biochemistry and molecular structure of an organism, as an important characteristic of ASD. ELISA, LC-MS/MS, and various biochemical assays have significantly shown an increase in oxidative stress factors such as 8-Hydroxy-deoxyguanosine, bisphenol A, 8-isoprostane, oxidized glutathione, and lipid peroxides in the cell lines of autistic groups as compared to control groups [14,15] and decreased levels of antioxidants such as glutathione, vitamins E, C, and A, etc. [16]. There has been a significant rise in mitochondrial dysfunction in the frontal lobe, temporal lobe, and cerebellum that suggests that lactate, pyruvate, creatine kinase, coenzyme Q10, and mitochondrial enzymes are directly related to energy metabolism dysfunction [9,17]. There is also evidence of immune dysregulation and persistent inflammation observed as a rise in cytokines (such as TGFβ2), HSP70, inflammatory proteins, and lipid mediators including cysteinyl leukotrienes in the serum samples of ASD patients [18,19]. Accompanied by the changes in fatty acid profiles, there has been a decrease in the activity of pyruvate kinase and hexokinase, with an increased concentration of the S-adenosylmethionine pathway members, causing systemic dysfunction [20]. Environmental and epigenetic factors such as bisphenol A exposure and alterations in gene expression that concern antioxidant processes, including SOD2 and GCLM alleles, point to their involvement in the pathophysiology of ASD [21,22]. In addition to that, several markers of oxidative stress, such as 10-F4t-NeuroP, 3-chlorotyrosine, and thioredoxin-related enzymes in ASD subjects’ plasma, have also been reported rather consistently [23]. These findings point in the direction of future studies developing site-specific antioxidant therapies, examining the mechanistic relations underlying oxidative stress and manifestations of ASD, and establishing biomarkers for early detection and individualized treatment.

Due to the imbalance between ROS and antioxidant systems, various tissues of the body can be damaged, including the brain [24]. Pivotal studies have suggested that autistic subjects experience increased oxidative stress markers, including lipid peroxidation products and protein carbonyls, along with DNA oxidation markers. If such an oxidative state exists at greater levels in brain areas of the PFC and temporal lobes concerning language and speech functioning, then it makes social and communication norms more difficult [10]. Increased oxidative stress can interfere with the function of mitochondria and neurotransmitters, which are both important in processing and learning language [25]. There is emerging literature demonstrating the benefits of antioxidant therapies in alleviating oxidative stress and enhancing the behavior of children diagnosed with ASD [26]. However, results are inconsistent, and more work is still needed. In people with ASD, considerable impairment of antioxidant systems has been demonstrated, wherein there is a decrease in glutathione levels, a key antioxidant that wards off oxidative stress to the cell. Glutathione is crucial in detoxifying ROS, in the maintenance of cellular integrity, and in the amelioration of neurodevelopmental processes. Research has shown that people with ASD generally show lower concentrations of reduced glutathione, which is the active form of glutathione that helps to mitigate stress, and the GSH/GSSG ratio in the cerebellum region of the brain indicates oxidative stress and weak protection at the cellular level [27]. The deficit of this glutathione discloses an insufficient ability to compensate for oxidative stress in the brain, something that could explain some neurodevelopmental features characteristic of autism. Oxidative stress is a vital component related to the development of ASD, which can alter the functioning of the central nervous system (CNS) and the gastrointestinal tract (GIT). This takes place with an elevation in lipid, protein, and DNA oxidation, along with the presence of inflammation and mitochondrial injury. These steps are linked with environmental risk factors, genetic predisposition, and lower antioxidant capacity. Studies show that oxidative stress markers are heightened in patients suffering from ASD, which suggests that oxidative stress may be one of the culprits of the pathophysiology of ASD, especially in terms of neuroinflammation hyperactivation, abnormal immune responses, and destruction of brain tissues.

Oxidative stress develops, as a rule, from the excessive production of ROS or, rather, the inability to remove these highly damaging intermediates and oxidative damage. Specific biomarkers referring to the exacerbation of oxidative stress related to ASD have been identified, which are discussed as follows.

### 2.1. Malondialdehyde (MDA)

Free radical attacks on lipids cause lipid peroxidation, which destroys cell membranes and produces MDA as a result. MDA levels often rise during oxidative stress, as one might expect. Research indicates that individuals with ASD have higher MDA concentrations in their urine and blood, suggesting increased oxidative stress and lipid peroxidation throughout their bodies [28,29].

### 2.2. 8-Hydroxy-2′-deoxyguanosine (8-OHdG)

8-Hydroxy-2-deoxyguanosine (8-OHdG) is one of the most developed oxidative DNA damage biomarkers, which results from the reaction between ROS and guanine bases of the DNA. An increased level of 8-OHdG identifies an increase in oxidative stress in the microenvironment of cells, thus demonstrating the level along with the degree of oxidation of DNA and the following genotoxic burden in the given tissues. Over the recent past, increased levels of 8-OHdG have also been reported in central (brain) and peripheral (blood/urine) tissue samples of ASD patients compared with those of their neurotypical peers. These results imply chronic oxidative damage to DNA in ASD, an occurrence that can trigger poor neuronal functioning, abnormal neurodevelopment, and the behavioral alterations commonly witnessed in ASD patients [11,30]. Moreover, the observation of excess 8-OHdG in decorated SUGs of the peripheral blood can possibly be used as a non-invasive measure of access to oxidative stress conditions in ASD. Moreover, the biomarker enhances the argument that systemic oxidative imbalance may have effects on the neural and peripheral tissues, which confirms the interdependence of the ASD pathophysiology by oxidative stress, mitochondrial dysfunction, and immune dysregulation [31,32].

### 2.3. Protein Carbonyls

The presence of protein carbonyls in biological systems has been linked to protein oxidation, as these compounds are created during the reaction of proteins with reactive carbonyl species. More recently, elevated levels of protein carbonyls have been noted in the brains of patients suffering from ASD, lending credence to the hypothesis that oxidative stress leads to protein degradation in these individuals [33,34].

### 2.4. Glutathione (GSH/GSSH)

Glutathione is a thiol-containing molecule, the primary and one of the most crucial antioxidants in the brain, helping to balance the oxidative stress system by countering it. The reduced glutathione (GSH) and oxidized glutathione (GSSG) ratio is an essential indicator of cellular oxidative damage. It also acts through working with glutathione reductase and peroxidase to maintain redox equilibrium. The postmortem brain samples of autistic subjects have demonstrated a reduced glutathione redox capacity in the cerebellum, signifying an elevated oxidative stress level affecting ASD patients [11,16,28].

**Figure 2:**
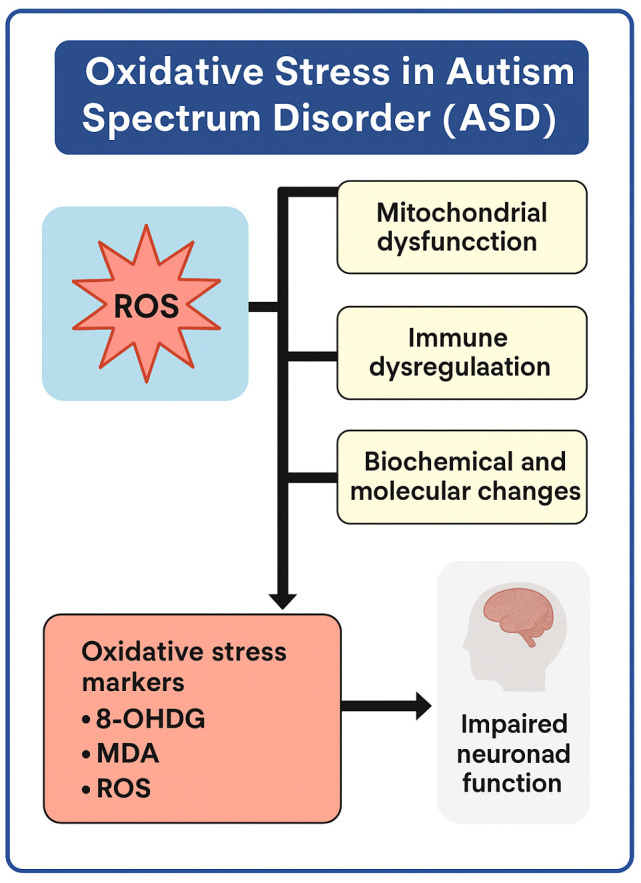
Role of oxidative stress markers and ROS in ASD-related biochemical, molecular, and neuronal changes in the brain. ROS-Reactive oxyfen species; 8-OHdG-8-Hydroxy-2-deoxyguanosine; MDA-malondialdehyde.

ASD, as a neurodevelopmental disorder, exhibits a wide range of phenotypic manifestations and a multifactorial nature. In its pathogenesis, an indispensable role of oxidative stress is anticipated according to empirical and theoretical evidence. Oxidative stress is a fundamental cellular process, and it entails the involvement of ROS. An increase in the levels of ROS, especially in mitochondria, disturbs organelle homeostasis and triggers the dysregulation of neurotransmitter metabolism, among other metabolic processes. These changes, which follow in these systems, along with immune dysregulation, lead to widespread biochemical and molecular illnesses. Interestingly, the biomarkers of oxidative stress, such as 8-hydroxy-2′-deoxyguanosine (8-OHdG), malondialdehyde (MDA), and ROS itself, are directly related to disturbances in neuronal functioning, which is very consistent with the cognitive impairment, speech problems, and behavioral abnormalities found in ASD (Figure 2).

## 3. Impact of Oxidative Stress on Speech and Language Development

Oxidative stress can affect the PFC and the temporal lobe, which are important for speech and language processing. If these areas are affected, then understanding and processing language become harder.

When the brain’s defense systems, such as the blood–brain barrier, meninges, and neuroimmune system in the form of microglia, are overstretched or compromised, it causes the release of free radicals, leading to oxidative stress. This can result in serious damage to vital areas such as the PFC, hippocampus, and amygdala. This damage can propagate neurodegenerative conditions such as Alzheimer’s disease and Parkinson’s disease alongside other neuropsychiatric and neurodevelopmental disorders such as depression, anxiety, schizophrenia, and autism by impairing cognition, memory, intellectual, social, and emotional regulation [35,36]. Oxidative damage is worsened by chronic stress and causes severe damage to the brain’s plasticity. Antioxidant defense systems such as superoxide dismutase, catalase, and glutathione help upregulate the Nrf2-ARE pathway. This upregulation leads to the activation of cellular defense mechanisms [37]. Because of these factors, treatments that target oxidative stress, such as antioxidants, are essential for preserving brain health. The impact of oxidative stress on the PFC is a reduction in the dendritic branching and connectivity, which ultimately leads to cognitive, emotional, and behavioral deficits present in autism. The existing reports do not explicitly point out the temporal lobe, but they do note oxidative damage in the hippocampus, which is associated with learning and memory, that may interfere with language functions in autism. Heightened levels of ROS, neuroinflammation, and the depletion of glutathione fuel these deficits, which indicate the importance of the efficacy of antioxidant therapies toward oxidative stress-induced brain dysfunctions in autism [38,39]. The temporal lobe and PFC, key regions for cognition, emotional control, and language, are compromised by oxidative stress. In ASD, oxidative alterations to the PFC lead to the disruption of a person’s executive functions, which include decision-making, impulse control, attention, social interaction, and spontaneous flexibility in adjusting to novel circumstances. Mitochondrial dysfunction, coupled with irregular glutamate signaling, further exacerbates the impairment of emotion regulation and behavioral flexibility, which are fundamental characteristics of ASD. Moreover, oxidative stress within the temporal lobe, including Wernicke’s area, compromises the ability to comprehend speech and hinders one’s capacity for verbal expression, resulting in speech delays and impaired auditory language processing [40]. These disabilities are frequently associated with sensory processing disorders as oxidative stress impacts hearing ability, making it challenging for people suffering from autism to filter, comprehend speech, and interpret surrounding sounds accurately [11]. Studies suggest that people with ASD have an increased concentration of ROS and a decreased reserve of antioxidants, thereby being easily susceptible to neuronal damage in certain vital areas of the brain. Hence, oxidative stress might be the reason for the prominent features of autism, such as language impairment, problems with social communication, and rigidity of thought. Some reports indicate that administering antioxidant medications may reverse the process of oxidative damage and subsequently improve the ability to process language, control higher mental functions, and regulate the senses in children with ASD [41].

## 4. Mitochondrial Dysfunction in ASD-Related Neurological Disorders

Mitochondria are crucial for producing energy within the cell. ASD is characterized by mitochondrial dysfunction that leads to decreased ATP production and an increase in ROS and reactive nitrogen species (RNS). This dysfunction originates from abnormalities in the electron transport chain (ETC) components and disturbances in calcium homeostasis, which increase oxidative stress and programmed cell death in neurons [42]. Neurodevelopmental processes, particularly those crucial for speech and language, may be impacted by oxidative stress and mitochondrial dysfunction. Increased oxidative stress in ASD children is shown by the significant reduction of glutathione (GSH) coupled with increased ROS, which has been correlated with communication deficits, specifically speech and language skills. This may result from injury to Broca’s and Wernicke’s areas or nonspecific developmental retardation due to oxidative injury of the neural tissues. In a paper published by Siddiqui et al., the authors have explored the increasing evidence of the link between ASD, a neurodevelopmental disorder that exhibits social communication deficits and repetitive behaviors, and abnormalities of the mitochondria [43]. Research has shown that individuals with ASD tend to be less physically active and to be prone to compromised energy metabolism and energy utilization when compared to the rest of the healthy population. This imbalance in energy metabolism is particularly reflected in the brain’s frontal and cerebellum regions, where there is diminished activity of the mitochondrial ETC complexes, as mitochondria are the epicenter of energy generation and are highly influenced by the individual’s physical activity. Because of that, there is a low output of ATP. Further, analyses of gene expression profiling have indicated the downregulation of the genes controlling energy metabolism within the mitochondria [44]. Moreover, there is increased oxidative stress, which indicates higher levels of cellular injury due to the excess ROS. In addition to that, there is evidence of altered mitochondrial DNA (mtDNA) copy number mutations and deletion in ASD patients, which suggests possible genetic factors responsible for the mitochondrial deficits [45]. Nevertheless, all these facts together have not fully elucidated the issue due to methodological inconsistencies and limited sample sizes, which make it unclear whether mitochondrial dysfunction is the cause or consequence of ASD. Expanding the sample size and utilizing advanced in vivo imaging modalities, such as near-infrared spectroscopy, would greatly enhance the understanding of the mitochondria’s role in the development of the disorder, as well as possible treatment strategies.

The complex issue of mitochondrial defects has become a key component of ASD. Research shows that approximately five percent of people suffering from ASD are considered to have met the criteria for mitochondrial disease (MD), which is much more than the general population’s prevalence of 0.01%. Even beyond the diagnostic criteria set for MD, a considerable number of individuals with ASD display biochemical evidence of mitochondrial damage, such as increased lactate and pyruvate levels, lowered carnitine, and ETC impairment, all of which indicate altered bioenergetics [46]. Clinically, ASD patients suffering from mitochondrial damage often have developmental regression, seizures, motor delays, and intestinal problems that point to the fact that mitochondrial damage affects both the brain and the body systems in people with ASD [9]. Neuroimaging as well as post-mortem research substantiate this link by showing that there is a perturbation in oxidative metabolism, decreased production of ATP, and increased oxidative stress in the ASD brain. Although genetic mutations explain 21% of ASD cases with MD, environmental factors, especially immune dysregulation and oxidative damage, are now seen more as contributing to secondary mitochondrial dysfunction. Additionally, in vitro studies show increased excretion of ROS and lowered antioxidant abilities among people with ASD, pointing to the increased vulnerability of mitochondria. Even though there is no concrete or definite mitochondrial-targeted therapy yet available, there are preliminary indications that affected individuals may benefit from carnitine, coenzyme Q10, and B vitamins, which enhance mitochondrial activity, thus improving symptoms. Further details are provided in Section 7. Further studies are needed to determine whether mitochondrial impairment leads to primary pathology in ASD or is simply a secondary effect, and what could be the most important elements of intervention using mitochondrial targets.

Moreover, mitochondrial dysfunction in conjunction with oxidative stress plays a significant role in various ASD-related neurological disorders in terms of its genetic impact and autism-associated developmental and cognitive symptoms (Table 1). Hence, some diseases involving mitochondrial dysfunction have been shown to correlate with ASD in terms of pathology and symptoms. For example, Smith–Lemli–Opitz Syndrome (SLOS) is a developmental disorder caused by a mutation in 7-dehydrocholesterol (7DHC), which leads to reduced cholesterol synthesis and energy production. This can impair proper brain development and may result in intellectual disability, a common symptom in ASD [47]. In addition, Helsmoortel–Van der Aa Syndrome (HVDAS) is considered a very rare neurodevelopmental disorder caused by an ANDP mutation, with possible manifestations such as developmental and intellectual delays, resembling ASD features. A small percentage of ASD patients (0.17%) also show a mutation in the ANDP gene [48]. Likewise, DiGeorge Syndrome (DGS) involves loss of function in the SLC25A gene, leading to mitochondrial dysfunction and energy imbalance. This disruption can slow down learning during childhood [49]. Similarly, the gene UBE3A, which is widely expressed in mitochondria and involved in brain development and synaptic plasticity, is linked to Angelman syndrome (AS). This creates an association between AS and ASD. Malfunction of this gene can hinder learning during neurodevelopmental stages [50]. Concerning ASD, Fragile X Syndrome (FXS) is another intellectual developmental disorder characterized by mutations in mitochondrial genes, including FMR1, which presents with neurodevelopmental and psychiatric challenges [51]. Rett syndrome, an X-linked neurodevelopmental disorder involving a dysfunctional MECP2 gene, is also commonly associated with ASD [52]. Cornelia de Lange Syndrome (CdLS) also exhibits autism-related features, such as impairments in verbal communication and a genetic abnormality involving TRMT61A protein mislocalization, ultimately affecting normal mitochondrial function [53]. Lastly, autism has also been linked to mutations or deletions in the SHANK3 gene, which have been reported in Phelan–McDermid Syndrome (PMS). These mutations are responsible for synaptic dysfunction in mitochondria [54].

## 5. Mitochondrial Dysfunction and Speech and Language Development

Disruption in speech and language development may occur due to mitochondrial dysfunction. Evidence shows that certain children with ASD and behavioral deficits in communication skills have low GSH levels alongside high ROS in mitochondria. This likely results from damage to the neural tissue processing language, which involves Broca’s and Wernicke’s areas, or generalized oxidative neural mitochondrial damage resulting in developmental delays. The genes associated with speech and language development can be upregulated or downregulated upon encountering oxidative stress and mitochondrial dysfunction. The generation of free radicals in the speech centers mentioned above could contribute to the degeneration or atrophy of these areas. This could result in severe impairment in the learning process of language or speech maintenance. Oxidative free radicals and inflammatory cytokines resulting from mitochondrial dysfunction also impact cognitive decline while verbal processing, thereby enhancing the brain’s vulnerability to ASD symptoms. Furthermore, dysfunctional mitochondria in these brain regions are responsible for the impaired or imbalanced generation of required energy for the propagation of vocal speech and language acquisition. Pregnant mothers consuming drugs or a diet rich in antioxidants have been reported to produce offspring with a better capacity to learn language from adults during the developmental period of sensory and motor learning of language [30,55].

## 6. Neuroinflammation in Autism Spectrum Disorder

Inflammation is a phenomenon common to both peripheral and central systems; however, when its epicenter is the brain, then it is regarded as neuroinflammation. Simultaneously, peripheral inflammation too often leads to neuroinflammatory conditions. Out of these, ASD has also been linked to this detrimental feature. This was reported to be associated with upregulated proinflammatory cytokines (IL-1, IL-6, IL-8, IL-12, IL-18, TNF, etc.) and downregulated anti-inflammatory cytokines (e.g., IL-4, IL-10, IL-13, TGF, etc.) [56]. These cytokines have the potential to cross the blood–brain barrier (BBB). The major brain regions in ASD prone to neuroinflammation are the cerebral cortex, hippocampus, amygdala, cerebellum, and white matter [57]. When these brain regions become overwhelmed by the mentioned inflammatory cytokines, then social, cognitive, language, and speech impairments come into play. This targeted action leads to a distorted lifestyle of ASD patients and a huge burden on caregivers. Other factors contributing to ASD-related neuroinflammation include microglial activation, autoimmune reaction, and genetic predisposition [58]. Even though these additional factors are indirectly related, they still play a significant role in comorbidity in ASD patients.

Furthermore, chronic neuroinflammation is seen in postmortem tissues of ASD patients’ brains, with microglial activation and high levels of proinflammatory cytokines reflecting a marker of persistent immune reaction [59].

### 6.1. Maternal Immune Activation-Mediated Neuroinflammation

**Figure 3:**
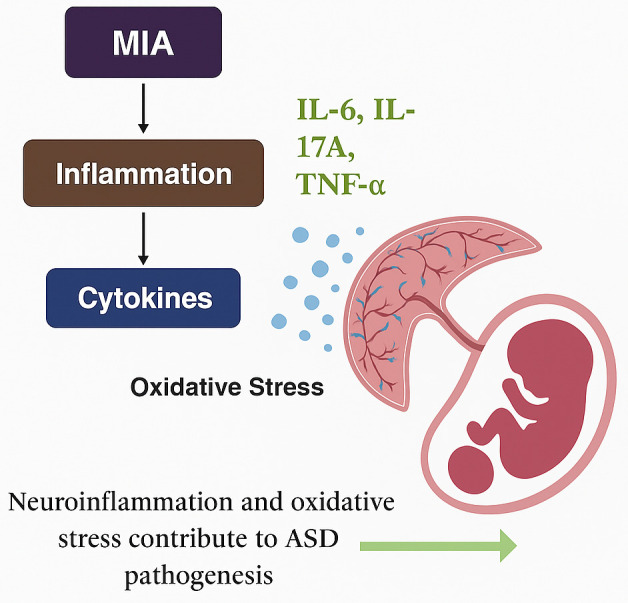
Role of oxidative stress and inflammation in maternal-induced ASD. MIA—Maternal immune activation, IL-interleukin, TNF-Tumor necrosis factor. **Light Pink Placenta & Fetus Illustration:** Depicts placental structure and developing fetus affected by the pathway. **Blue Dots:** Symbolize oxidative stress factors crossing the placenta.

Maternal immune activation (MIA) is an environmental factor that influences the risk of ASD and occurs when a mother develops an immune response during pregnancy because of an infection, autoimmune disease, or chronic inflammation [60]. This response triggers the secretion of proinflammatory cytokines, including IL-6, IL-17A, and TNF-α, which can cross the placenta and disrupt fetal brain development [61]. MIA can disrupt neurogenesis, synaptic growth, and neurotransmitter balance and would result in ASD-like traits [62] (Figure 3). Social emulation, repetitive behaviors, and abnormalities of sensation are the most frequent types of such behaviors. In addition, prenatal brain oxidative stress, which serves as one of the principal etiological factors of autism, is associated with significant mitochondrial damage that further worsens the neuronal assault and amplifies the central neural circuit’s destruction [63]. Emerging evidence from studies conducted on both animals and humans indicates that inflammatory processes caused by maternal immune activation can potentially be modified in a way that reduces the chances of suffering from ASD or its symptoms. Experimental studies on animals reveal that the overstimulation of proinflammatory cytokines and their receptors at critical stages of fetal brain formation can damage the cortical structure and result in permanent behavioral disturbances such as those exhibited by patients with ASD.

### 6.2. Microglial Activation

Microglia are resident immune cells of the brain, responsible for defending from brain infections and clearing cellular waste. Upon activation, they also trigger the release of inflammatory mediators, paving the way to neuroinflammation. Every case of ASD has a common manifestation of neuroinflammation. This also involves the brain microglial cells, leading to chronic inflammation and oxidative damage. In this regard, microglial activation leads to oxidative stress via the NADPH oxidase (NOX) enzyme, more precisely the NOX2 isoform being overexpressed on microglial cells, facilitating the production of the superoxide free radical [64]. The heightened oxidative stress can further lead to microglia-mediated neuroinflammation and vice versa, propagating the cycle to continue. Such processes are regarded as neurotoxicity and are detrimental to developing brains [65].

Microglia also express GABA receptors, and hence, dysregulated expression of the receptors results in abnormal cortical development and the underdevelopment of GABAergic neurons, which are crucial for inhibitory neurotransmission, and, ultimately, an imbalance in excitatory/inhibitory signaling [66]. These activities can be a potential contributor to the pathogenesis of ASD.

## 7. Antioxidant, Mitochondrial, and Anti-Inflammatory Therapy

Antioxidants such as polyphenols and carotenoids have been widely investigated preclinically; however, translation to clinical levels is minimal. Exogenous antioxidants are often a good replacement when endogenous ones have been depleted due to any reason, for example, mitochondrial dysfunction or a dysregulated immune system. Antioxidant therapy could also be used as adjuvant therapy or monotherapy when required. However, these plant-derived therapies, including polyphenols, carotenoids, etc., have not been widely tested for their toxicity, thereby limiting their use clinically. On the other hand, the dose at which these preclinically tested substances show efficacy might not necessarily elicit the same response. These drawbacks emphasize the further investigations of antioxidant substances at in vitro and in vivo levels. Some of the extensively recognized naturally occurring antioxidants include vitamins, flavonoids, anthocyanins, carotenoids, etc. [27,67].

Some other substances, such as thiol antioxidants (N-acetyl cysteine), free radical scavengers (vitamin C), sulforaphane, and coenzyme Q10, also seem to reduce tissue oxidation, inflammation, and complex autism symptoms [68]. Here, vitamin C acts by donating an electron to neutralize free radicals and also helps in initiating another antioxidant, vitamin E. Coenzyme Q10 is a well-known, naturally produced biochemical cofactor in the human body, having a role in antioxidant mechanisms, and is also available as a supplement when there is a deficiency. Furthermore, antioxidants affect several signaling pathways. For example, resveratrol targets NADPH oxidase to reduce ROS generation. In the category of carotenoids, beta-carotene, lutein, and lycopene are found in fruits and vegetables; hence, consumption by children might slow down the ASD-related oxidative stress. Dry fruits such as walnuts and almonds are also high sources of antioxidants in the form of omega-3 fatty acids. Other polyphenols including curcumin, ellagic acid, gallic acid, ferulic acid, and vanillic acid can also be investigated and formulated for their antioxidant action against autism [67].

Additionally, in the context of mitochondria, mitoquinone mesylate (mitoQ) and 2,2,6,6-Tetramethylpiperidine 1-oxyl (mitoTEMPO) have been reported to act as mitochondria-targeted antioxidants. Here, mitoQ functions by scavenging ROS; on the other hand, mitoTEMPO is an SOD-mimicking compound, thereby reducing the overall oxidative damage [69]. Interestingly, peptides have been designed to navigate proteins or other therapeutic molecules towards the mitochondria to fix the mitochondrial dysfunction. Examples of mitochondria-targeting peptides include Szeto–Schiller peptides, self-assembled peptide nanomaterials, mitochondria-penetrating peptides, etc. [70,71].

In terms of anti-inflammatory agents to be repurposed or to potentially be used for ASD, there is a long list, but some of the important ones are corticosteroids such as prednisolone, NSAIDs including celecoxib, etc. Tetracycline, like minocycline, has also shown promising immunoregulatory and anti-inflammatory actions [72]. Alternatively, several natural anti-inflammatory agents have shown effects at the preclinical stage and can be extended to the clinical level. For example, resveratrol, luteolin, and quercetin have been investigated to reduce proinflammatory cytokines in animal models of ASD. Some of the clinical studies have also shown that GSH, Vitamin C, and Palmitoylethanolamide can alleviate inflammatory conditions in autistic patients [73]. Furthermore, immune-regulated improvement in inflammatory conditions through intravenous immunoglobulins and B-cell targeted therapy with rituximab has also been beneficial to some extent. Additional research on other inflammatory signal blocking agents, such as anakinra (IL-1β inhibitor), and inflammasome blockers, such as colchicine, etc., will open the door for further potential anti-inflammatory ASD therapies [74].

Of note, IL-6 or IL-17A blocking during pregnancy has been shown to have a therapeutic benefit in reducing such effects through experimental intervention. In one notable study, Choi and collaborators (2016) demonstrated that the administration of neutralizing IL-17A monoclonal antibodies in pregnant mice exposed to MIA effectively prevented cortical malformations and limited the appearance of ASD-like behavior in the offspring [62]. Similarly, IL-6 signaling blockade has proven to be able to reduce behavioral deficits and neurodevelopmental abnormalities caused by prenatal inflammation [63]. For example, some interventions such as anti-inflammatory celecoxib and immunoregulatory prednisolone during pregnancy have been shown to block IL-17A and IL-6 cytokines, which seems to diminish the chances of the child exhibiting ASD-like behaviors [72].

These findings show that inflammation- and oxidation-focused therapies, including anti-inflammatory agents, probiotics, and nutritional antioxidants, can be used during pregnancy to potentially decrease the risk of ASD and support better development in affected children.

## 8. Environmental Factors in Autism Spectrum Disorder

Environmental sources have also been reported to trigger ASD-related pathophysiology. The exposure to these environmental culprits could be either to the pregnant mother or the newly born infant. Either way, it can adversely impact the neurodevelopmental process of the child. Environmental exposures in the form of heavy metals such as mercury, arsenic, and lead; pollution with particulate matter; and even maternal factors, can enhance ROS production, which together build oxidative stress and negatively affect early brain development, which is critical for language development [75]. Additionally, certain pesticides, maternal obesity, immune dysregulation, and diabetes mellitus can also predispose the fetus to etiological factors of ASD [76]. Moreover, infections and certain medications including antidepressants and antiepileptic drugs consumed during pregnancy can produce hazardous results to a child’s brain if they exhibit neurotoxicity [77]. Nutritional deficiency of certain essential minerals and vitamins can lead to severe consequences, such as diminished progression of the normal neurodevelopmental process. There is growing evidence that oxidative stress, mitochondrial dysfunction, and immune dysregulation are key components in the environmentally generated pathophysiology of ASD. Studies show that ASD patients exposed to a deleterious environment have increased chances of oxidative stress leading to damage to lipids, proteins, and DNA in important parts of the brain responsible for speech, social behavior, memory, and motor activities [78]. It is widely known that the loss of function of the ETC, changes in mitochondrial gene expression, and reduced ATP synthesis occur with neurodevelopmental disabilities when mothers or children are exposed to harmful surroundings. Together, these abnormalities point to an underlying structure for ASD that may arise from preexisting environmentally induced factors. Although the specific causal mechanisms are unknown, this collection of literature amplifies the need for deliberately focused therapies to reverse the damage sustained by the mitochondria, decrease oxidative injury, and alter the level of inflammation in the brain to improve the condition of individuals with ASD.

## 9. Gut–Brain Axis in Autism Spectrum Disorder

The gut microbiome has been reported to impact brain health to cause many neurological disorders, including ASD. The gut bacteria have the potential to release neurotransmitters and hence influence brain function [79]. These microorganisms also influence the immune system and inflammatory process, thereby worsening the patient’s capacity to defend against the harmfulness of the bacteria. Furthermore, dietary imbalance can also destroy the flora of the gut, and even useful bacteria may lose their abundance [80]. This could aggravate the brain’s protective mechanisms, and hence, more complications with dysbiosis will arise. These disturbances could appear even in the form of oxidative stress as well, as there will be energy metabolism dysregulation propagated by mitochondrial dysfunction of the gut. Children with immunodeficiency and those who often suffer from diarrhea may lose essential nutrients and microbiome and, hence, are more prone to gut-related brain dysfunction and even downregulation of the genes responsible for social communication and speech development [81]. Hence, proper maintenance of a diet moderately cooked and loaded with essential nutrients is necessary for the smooth functioning of the gut–brain axis.

In addition to food, certain drugs can also hurt the gut flora, resulting in repetitive or restrictive behavior in children. Probiotics, prebiotics, and synbiotics can have a positive influence; on the other hand, antibiotics can initiate side effects. Interestingly, ASD patients taking medications for symptomatic relief should be cautious in preventing drug interactions and drug-induced microbial flora deteriorations. Additionally, the maternal gut flora can also influence the offspring depending on the placental permeability of drugs or foods [82].

In a recent report published in Nature Neuroscience, a temporal and bidirectional relationship between microbiome composition and ASD phenotypes was found. These patterns were depicted in age-matched and sex-matched cohorts through dietary profiles, metabolomics, and inflammatory cytokines [83]. This further strengthened the concept of the gut–brain axis role in ASD symptoms.

Connecting the gut relations with brain oxidative stress, mitochondrial dysfunction, and neuroinflammation, it has long been hypothesized that an unbalanced diet or dysbiosis may have potential contributions to generating oxidative stress in the brain. High-fat diets rich in cholesterol and LDL are the risky ingredients that play a role in brain oxidative damage and immune-dysregulated neuroinflammation. These factors suggest a strong gut–brain axis role in ASD.

## 10. Genetics and Epigenetics in Autism Spectrum Disorder

The early stage of brain development is highly influenced by environmental factors, which determine the expression levels of genes responsible for speech, language, communication, intellectual ability, and social behavior. These changes in the gene parameters are regarded as epigenetics and can be considered a novel trend and target in ASD research. Histone modifications with histone deacetylases and DNA methylation of genes with methyltransferases can lead to the development of autistic traits and social isolation behavior.

In the context of the present review, oxidative stress and proinflammatory cytokines have been noted to alter histone acetylation and DNA methylation, predisposing to the genetic changes associated with ASD-related pathology. Hence, these epigenetic changes can be reliable pathways to target ASD with the purpose of therapeutic interventions. Furthermore, epigenetic alterations in mitochondrial DNA have been investigated to have a greater influence on initiating the ASD-related dysfunction of energy metabolism. This can delay or halt the energy production necessary for the normal functioning of the brain in intellectual tasks such as focus, attention, thinking, and even the communication and development of speech and language centers in the brain.

Furthermore, an opinion piece has discussed gene polymorphisms resulting from dietary variation. This variation could be in the form of polymorphisms in the methylenetetrahydrofolate reductase (MTHFR) C677T gene in ASD children who lacked folic acid fortification in their diet. Additionally, microRNA dysregulation, transgenerational inheritance, increased expression of Tet methylcytosine dioxygenases, hypomethylation in the MAP8KIP3 or NALP1L5 gene, and downregulation of DNA methyltransferase 1 were also found to be associated with autism-related disorders [84].

Several other genes that play a role in the pathogenesis of ASD include SHANK3, MECP2, YTHDF, FRMPD4, FOXP2, PTCHD, HOX, CHD8, etc. These genes have been widely reported to play roles in neurodevelopmental features. For example, FOXP2 has a role in speech and language development, where it is downregulated in children with ASD speech difficulty. Similarly, either the upregulation or downregulation of these genes can have a role in features such as nerve cell communication, intellectual development, neurotransmitter release, and others [85,86,87].

Polymorphisms of genes that are part of antioxidant pathways, including glutathione peroxidase (GPx) and superoxide dismutase (SOD), are just a couple of genetic factors that make some people more susceptible to oxidative stress [68].

## 11. Recent Improvements in the Evaluation and Management of Autism Spectrum Disorders: Diagnosis and Treatment

ASD refers to a wide range of neurodevelopmental disorders defined by deficits in social interactions, communication skills, and other sensory behaviors. Increased efforts in biomarker discovery may allow for the more accurate sub-typing of ASDs with specific targeted interventions (Table 2).

**Table 2:** Gene mutations and genetic targets involved in ASD-related behavioral and neurological symptoms and possible treatment options.

Gene Function	Genetic Variant/Mutation Type	Behavioral Changes	Neurological Effects	Molecular and Cellular Alterations	Possible Treatments
Dup 15q11-q13 [88]	Chromosome 15 portion duplication	Reduced social interaction, fewer vocalizations, developmental delays	Rigid behavior patterns	Impaired serotonin signaling, enhanced spine density	Behavioral and speech therapy, no specific gene manipulation therapy
TBR11 [89]	Missense mutation	Reduced social interaction, altered food preference learning, intellectual disability	Rigid behavior, learning difficulties	Abnormal axonal links in amygdala, reduced NMDAR activity	Lithium chloride, D-cycloserine rescues synaptic function in Tbr1 mutant mice
FMR1 [90]	Trinucleotide repeat expansion	Reduced social interaction, fewer vocalizations	Repetitive movements, hypersensitivity to sound, learning issues	Disrupted neural connectivity, increased mGluR activity, and replaced synaptic plasticity	Pirenperone reduces hyperactivity in Fmr1 KO mice
SHANK2 [91]	Frameshift mutation	Reduced social behaviors, hyperactivity	Repetitive movements, cognitive difficulties	Impaired excitatory, synaptic dysfunction	Downstream of SHANK2 such as NMDA receptor and ERK pathway can be targeted
SHANK3 [92]	Frameshift mutation	Reduced social behaviors	Excessive grooming, anxiety	Insufficient striatal activation and decreased AMPAR function	IGF1 enhances long-term potentiation and motor function in SHANK-deficient mouse model
SCN1A [93]	Missense mutation	Reduced social behaviors	Seizures, learning deficits, grooming repetition	Reduced activity of inhibitory interneurons	Clonazepam improves neurobehavioral activities by targeting the Scn1 gene in PFC
CNTNAP2 [94]	Deletion	Reduced social behaviors, intellectual slowness, fewer vocalizations	Hyperactivity, repetitive grooming, seizures	Reduced interneuron population, disrupted neuronal migration	Risperidone and Oxytocin alleviate repetitive behavior in Cntnap2 −/− mice
TSC1, TSC2 [95]	Loss-of-function mutation	Reduced social interaction, increased vocalizations	Enhanced repetitive behaviors, coordination difficulties, cognitive impairment	Enlarged brain size, overactive mTOR signaling, impaired autophagy	Rapamycin targets mTOR (overactivated by Tsc1 and tsc2) and hence decreases ASD neuropathology
PTEN [96]	Frameshift mutation	Reduced social interaction	Learning impairment, seizures, anxiety	Abnormal neuronal growth, PI3K pathway overactivation	Rapamycin improves social and stereotypic behavior in Pten KO mice
NLGN3 [97]	Point mutation	Reduced social interaction, fewer vocalizations	Increased motor activity	Increased mTOR/Akt activation, impaired GABAergic transmission in striatal neurons, synaptic dysfunction	There are no specific drugs to target Nlgn3, but rapamycin can be tested
NRXN1A [98]	Exonic deletion	Reduced social behaviors, aggression	Motor learning issues, sensory processing deficits, spatial learning deficits	Lowered glutamate transmission, decreased synaptic density	No specific drugs, but extensive research is ongoing to target Nrxn1
MECP2 [99]	Loss-of-function mutation	Reduced social interaction	Repetitive movements, motor deficits, seizures	Synaptic dysfunction, increased microglia activation, BDNF activation	IGF1, Clenbuterol, Fingolimod can enhance neuronal plasticity by targeting Mecp2
CX3CR1 [100]	Deletion	Reduced social interaction	Impairment in learning and memory, anxiety	Impaired synaptic pruning, overactive microglia, neuroinflammation	Microglial modulators, E6130 as anti-inflammatory, and AZD8797 to inhibit CX3CR1
CHD8 [101]	Loss-of-function mutation	Social deficit, repetitive behavior	Repetitive grooming, increased brain size, learning impairment	Disrupted neuronal differentiation, abnormal cortical development	Fluoxetine partially restores neurogenesis in CHD8-ablated mice
TCF4 [102]	Point mutation	Restlessness	Abnormal neuronal migration and excitability	Impaired neuronal plasticity, altered brain connectivity	Nicradipine improves learning, memory, and restlessness in TCF4 +/− mice
EIF4E KO [103]	Missense mutation	Reduced sociability	Self-grooming, contextual fear memory	Dysregulated protein synthesis, abnormal synaptic function	Mnk inhibitors dephosphorylate EIF4E to downregulate it

It is widely accepted that ASD stems from multiple causes, both of genetic as well as environmental origin. Newer research suggests the contribution of monogenic mutations (e.g., SHANK3, FMR1, MECP2) and polygenic interactions that result in synaptic dysfunction and other neurodevelopmental insults [104]. These genetic challenges could have possible links to the mentioned features of ROS, impaired energy metabolism, and inflammatory cytokines. Genetic analysis of ASD-specific genes allows the identification of risk-associated variants. Hence, the early detection and selective targeting of these genes for their upregulation or downregulation might be a significant step towards the evaluation and management of ASD. Maternal infections, toxin exposure during prenatal stages, and inadequate nutrition are other factors that aggravate the risk of ASD, indicating the gene–environment interaction [105]. Therefore, confinement and avoidance of the mother during pregnancy and even after the birth of the child from these environmental hazards is necessary to reduce the chances of the child being prone to ASD pathology. Furthermore, the development and refinement of technologies geared towards diagnosis have made quantitative improvements in the accuracy of identifying patients with conditions and implementing relevant therapies in those patients. Commonly used neuroimaging techniques including fMRI, DTI, and PET scans aid in the diagnosis of structural and functional abnormalities within the brain tissues of an ASD patient [106]. These approaches to depict brain regions specific for intellectual, communication, and vocal centers are crucial in uncovering brain atrophy, apoptosis, the deposition of certain plaques, or changes in symmetry impacted by phenomena such as oxidative stress, mitochondrial dysfunction, and neuroinflammation. All these outcomes could be contributing to the determination of ASD-related pathology and possible treatments to halt or reverse it. Moreover, AI-based screening and eye-tracking devices are other non-invasive modes of assisting in the early steps of ASD identification, enabling faster therapeutic actions to be taken [107]. Until recently, both behavioral interventions, including Applied Behavior Analysis (ABA) and social skills training, and their challenging techniques have always been the gold standard therapies for ASD [108]. Still, antipsychotic medications, notably risperidone and aripiprazole, are also commonly used to treat the violent and disruptive behavior patients may have [109]. Additionally, transcranial magnetic stimulation (TMS) and transcranial direct current stimulation (tDCS) are notable attempts at treating the abnormal neural circuits if distorted in ASD subjects [110]. Furthermore, therapies that focus on biofeedback try to improve self-regulation in patients suffering from ASD. There are also new virtual reality and AI-powered cognitive therapies that are more tailored to the patient’s needs. Diet and nutrition have recently become a focus as to what could prove useful in treating ASD. Research has shown promising effects on gluten- and casein-free diets, omega-3 supplementation, and targeted gut microbiome therapies to reduce symptoms related to ASD [111]. Many researchers have started paying attention to the role of the gut in the brain and vice versa, resulting in studies focused on probiotics, prebiotics, and fecal microbiota administration or ingestion as possible supplementary therapies for ASD [112]. Precision medicine, gene editing technologies (CRISPR-Cas9), and stem cell-based prospects have the potential for precision treatment and are where future research will be directed [113]. Additionally, more needs to be accomplished to encourage international cooperation, early intervention access, and public education to correct the imbalance in ASD diagnosis and treatment.

## Figures and Tables

**Table 1:** Role of mitochondrial dysfunction in neurological disorders and their association with ASD.

Syndrome	Mitochondrial Process Affected	Energy Metabolism Impact	Sample Source	Gene/Protein Involvement	Mitochondrial Impact	Neurological and Developmental Effects
Smith–Lemli–Opitz Syndrome (SLOS) [47]	Mitochondrial function and cholesterol metabolism	Disturbance of cholesterol processing, energy dysregulation	Fibroblasts from affected individuals	DHCR7	Accumulation of mitochondria dysfunctional substances	Developmental delay, weak muscle tone, behavioral differences
Helsmoortel–Van der Aa Syndrome (HVDAS) [48]	Cellular respiration efficiency	Deficiency of energy to operate cells	Fibroblasts from affected individuals	ADNP	Suppressed mitochondrial oxygen utilization, decreased production of ATP	Autism-like characteristics, retarded brain development
DiGeorge Syndrome (DGS) [49]	Mitochondrial transport and ion balance	Faulty mitochondrial integrity, increased oxidative load	Fibroblasts from patients and mouse models	SLC25A1, SLC25A4	Disruptions in mitochondrial carrier proteins, disturbances in calcium homeostasis	Deficits in learning, increased risk of psychiatric diseases such as schizophrenia
Angelman Syndrome (AS) [50]	Gene regulation and electron transport	Low levels of ATP production, mitochondrial shortages	Fibroblasts and hippocampal cells from UBE3A mutant mice	UBE3A	ETC complex III dysfunction, irregular expression of mitochondrial genes	Serious lag of mental development, epilepsy, movement disorders
Fragile X Syndrome (FXS) [51]	Mitochondrial structure and dynamics	A loss in cellular energy and oxidative stress	Brain tissue from FMR1 knockout mice	FMR1, MFN1, MFN2, OPA1	Loss of fusion protein levels, abridged ATP production, and lesser action in ETC complexes I and II	Impaired cognitive abilities, disrupted synapses in neurons, neurodegeneration
Rett Syndrome (RS) [52]	Mitochondrial structure and oxidative balance	More oxidative stress, poor energy consumption	Brain tissue from MECP2 knockout mice	MECP2	Mitochondria having aberrant morphology, long length of mitochondria	Motor impairment, intellectual disability, the likelihood of a seizure
Cornelia de Lange Syndrome (CdLS) [53]	Mitochondrial protein synthesis	Deficient mitochondrial protein synthesis	Skin fibroblasts from ASD patient	TRMT61A, MRPS22	Ribosomal mitochondrial defects and compromised complexes of ETC I, III, and IV	Height deficiencies, intellectual disabilities, unusual facial appearance
Phelan–McDermid Syndrome (PMS) [54]	Electron transport chain performance	Reduced supply of ATP, harm by ROS	Oral samples from PMS patients	SHANK3	Nonfunctioning complexes I and IV, and imbalance in the energy schedule of mitochondria	Learning disorders, deficit in neuronal signaling

## Data Availability

No new data were created or analyzed in this study.

## Abbreviations

ASD: Autism Spectrum Disorder; PFC: Prefrontal Cortex; ROS: Reactive Oxygen Species; ETC: Electron Transport Chain; MDA: Malondialdehyde; 8-OHdG: 8-Hydroxy-2′-deoxyguanosine; GSH: Reduced Glutathione; GSSG: Oxidized Glutathione; CNS: Central Nervous System; GIT: Gastrointestinal Tract; mtDNA: Mitochondrial DNA; MIA: Maternal Immune Activation; IL: Interleukin; TNF-α: Tumor Necrosis Factor-alpha; BBB: Blood–Brain Barrier; TGF-β: Transforming Growth Factor-beta; HSP70: Heat Shock Protein 70; ATP: Adenosine Triphosphate; SOD: Superoxide Dismutase; GCLM: Glutamate-Cysteine Ligase Modifier Subunit; NAC: N-Acetyl Cysteine; DTI: Diffusion Tensor Imaging; fMRI: Functional Magnetic Resonance Imaging; PET: Positron Emission Tomography; TMS: Transcranial Magnetic Stimulation; tDCS: Transcranial Direct Current Stimulation; ABA: Applied Behavior Analysis; FMR1: Fragile X Mental Retardation 1 Gene; SHANK3: SH3 and Multiple Ankyrin Repeat Domains 3; MECP2: Methyl-CpG Binding Protein 2; CHD8: Chromodomain Helicase DNA Binding Protein 8; PTEN: Phosphatase and Tensin Homolog; SCN1A: Sodium Voltage-Gated Channel Alpha Subunit 1; CNTNAP2: Contactin Associated Protein-Like 2; NLGN3: Neuroligin 3; TSC1/2: Tuberous Sclerosis Complex 1 and 2; BDNF: Brain-Derived Neurotrophic Factor; IGF-1: Insulin-like Growth Factor 1; CRISPR: Clustered Regularly Interspaced Short Palindromic Repeats.
